# Association between uric acid and brachial-ankle pulse wave velocity: secondary analysis of data from a cross-sectional study

**DOI:** 10.1038/s41598-020-59391-8

**Published:** 2020-02-10

**Authors:** Faxin Luo, Chaozhou Zhuo

**Affiliations:** Emergency Department, The People’s Hospital of Longhua, Shenzhen, China

**Keywords:** Biomarkers, Cardiology, Diseases, Endocrinology, Health care

## Abstract

At present, the association between uric acid (UA) and brachial-ankle pulse wave velocity (baPWV) has not been well clarified. This study is the second analysis based on a cross-sectional study. 912 participants (average age is 51.5 ± 9.6 years) who underwent medical health examinations were included in this study, UA levels and baPWV were measured. Participants were divided into four groups according to UA levels (Quantile 1: 2.00–4.10 mg/dL; Quantile 2: 4.20–5.20 mg/dL; Quantile 3: 5.30–6.00 mg/dL and Quantile 4: 6.10–9.80 mg/dL), and the differences of baPWV between the four groups were compared. Univariate analysis showed a positive correlation between UA and baPWV [(Quantile 2 vs Quantile 1: 8.85 (−36.05, 53.75); Quantile 3 vs Quantile 1: 60.32 (13.22, 107.42) and Quantile 4 vs Quantile 1: 80.34 (36.19, 124.49)]. After adjusting for confounding factors, the positive correlation between UA and baPWV still exists [(Quantile 2 vs Quantile 1: −9.92 (−60.16, 40.32); Quantile 3 vs Quantile 1: 82.34 (4.00, 160.68) and Quantile 4 vs Quantile 1: 143.13 (0.75, 285.51)]. Furthermore, curve fitting showed that UA and baPWV had a non-linear positive correlation. In conclusion, elevated UA were associated with baPWV, suggesting that UA could be used as a predictor of atherosclerosis.

## Introduction

With the continuous development of social economy and the change of people’s eating habits, cardiovascular disease has become the main cause of death worldwide^1–4^, especially the number of cardiovascular diseases has been increasing in recent years, this trend will be more pronounced in the future. As is known to all, cardiovascular disease has the clinical characteristics of high incidence, high mortality and high disability rate^5,6^. Once cardiovascular disease occurs, it will bring a huge economic burden to patients and national health security system. Therefore, early identification of cardiovascular risk factors and timely intervention is the focus of clinicians and medical and health institutions.

Previous studies have shown that atherosclerosis is an important pathological of cardiovascular disease^7–9^, but early atherosclerosis is lack of specific manifestations, and patients do not have obvious clinical symptoms, thus atherosclerosis is easy to be ignored in its early stages^10^. Therefore, how to identify arterial stiffness early is an important strategy to prevent cardiovascular diseases. With the gradual development of medical equipment and the continuous improvement of people’s attention to cardiovascular diseases, the technology of evaluating arterial stiffness by non-invasive method has become the focus of clinicians^11,12^. Brachial-ankle pulse wave velocity (baPWV)^13–16^ is the measurement of pulse wave velocity between brachial artery and ankle artery, which mainly reflects the stiffness of the vascular wall of peripheral large and middle arteries^17^. The larger the baPWV value, the higher the degree of arterial stiffness, and then the greater risk of cardiovascular diseases. At present, baPWV has been used as a non-invasive and important index to judge the degree of arterial vascular wall damage.

Uric acid (UA) is a feeble organic acid, which is the final decomposition product of purine nucleotides in blood circulation^18–20^. Previous studies have shown that UA may promote oxidative stress^21,22^, which might be related to the increase of oxygen free radical, nitric oxide and myeloperoxidase. In recent years, a series of reports have advocated that elevated UA are key hazard factors for the existence and development of cardiovascular diseases^23,24^. A prospective cohort study included 466 patients with ST-segment elevation myocardial infarction (STEMI) and UA level was measured. The results showed that patients with elevated UA had higher in-hospital mortality (OR 1.82, 95% CI 1.15–2.86)^25^. A meta-analysis of 33 studies involving 427 917 participants showed that elevated UA was significantly associated with heart failure, all-cause mortality and cardiovascular mortality^26^. Similarly, on another meta-analysis, the authors discovered that higher UA was closely related to atrial fibrillation in both cross-sectional and cohort studies^27^.

However, the association between UA and baPWV is still controversial^28,29^, and it is not clear whether they share some common mechanisms in the process of arterial stiffness. An investigation in South Korea achieved that UA level was associated with baPWV in males (p < 0.001) and in females (p = 0.04)^28^. In contrast, another study noticed that UA level was not connected with baPWV^29^. Therefore, our study targeted to inspect the association between UA and BAPWV based on a cross-sectional study.

## Methods

### Study participants

All data used in this research can be downloaded freely and without restriction from Dryad Digital Repository (https://datadryad.org/stash). The data used in this study are from a cross-sectional study of Japanese population (Fukuda, Takuya *et al*. (2014), Data from: Association between serum γ-glutamyltranspeptidase and atherosclerosis: a population-based cross-sectional study, Dryad, Dataset, 10.5061/dryad.m484p)^30^. Briefly, 912 participants who underwent health check-ups in Murakami Memorial Hospital from March 2204 to December 2012 were included. Exclusion criteria: (1) participants treated with hormones; (2) participants using oral contraceptives; (3) participants who tested positive for hepatitis B virus; (4) HCV-positive participants; (5) participants in pregnancy (women); (6) ankle-brachial index (ABI) value less than 0.95. Current research is the secondary analysis based on a previous study, thus it is not necessary to provide participants’ informed consent and approval by the ethics committee.

### baPWV, UA and other variables

It is necessary to point out that Fukuda and his colleagues completed the entire process of data collection (including the measurement of baPWV, UA and other variables, which has been described in previous studies^28^. In order to enable researchers or readers to grasp the measurement process noticeably, we briefly describe the measurement instruments and procedures here. Fukuda and his colleagues used the automatic waveform analyzer (Colin Medical Technology, Komaki, Japan) to measure the values of baPWV and ABI. This non-invasive method uses a cuff oscilloscope suitable for the arm and ankle to measure the pulses of the brachial and posterior tibial arteries of the participants. Before baPWV measurements, participants had to rest in a supine position at room temperature of 25 °C for 5 minutes. The electrodes were placed on both wrists of the participants, and the heart sound microphone was placed on the left edge of the sternum of the participants. Variables and formulas for calculating BaPWV (cm/s) have been described in Fukuda *et al*. studies^28^.

In addition, participant information downloaded from Dryad Digital Repository includes age (year), body mass index (BMI), systolic blood pressure (SBP), diastolic blood pressure (DBP), aspartate aminotransferase (AST), alanine aminotransferase (ALT), Log2glutamyltranspeptidase (Log2GGT), fasting blood glucose (FBS), total cholesterol (TC), triglycerides, high density lipoprotein (HDL), low density lipoprotein (LDL), uric acid (UA), alcohol consumption, estimated glomerular filtration rate (eGFR), ankle brachial index (ABI), sex, smoking status, Ex-Smoker, regular exerciser, fatty liver and menopausal status. According to Fukuda and his colleagues, venous blood was taken from the anterior elbow veins of the participants after 8 hours of fasting. The samples were gathered in a silicified glass tube containing sodium fluoride. After the venous blood was collected, the plasma and serum samples were centrifuged and stored at −80 °C. Professionally trained technicians performed abdominal examinations with Aloka SSD-650CL (Aloka Co, Ltd, Tokyo, Japan). The diagnosis of fatty liver was based on the results of abdominal ultrasound. In addition, standardized questionnaires were used to investigate participants’ basic information, including age, sex, smoking, drinking, exercise and menopausal status.

### Statistical analysis

In this study, participants were divided into four groups according to the level of UA, (Quantile 1: 2.00–4.10 mg/dL; Quantile 2: 4.20–5.20 mg/dL; Quantile 3: 5.30–6.00 mg/dL and Quantile 4: 6.10–9.80 mg/dL), and the differences of baPWV between the four groups were compared. The assumption of normality of distribution was examined using Shapiro-Wilk test. Considering the partial distribution of glutamyltranspeptidase (GGT), we convert GGT into log_2_ transformed, namely log_2_GGT.

For continuous data with normal distribution, variables are represented as mean ± standard deviation, and least significant difference (LSD) variance analysis was conducted to evaluate the differences between groups. For categorized data, variables are represented as number and percentages (n, %) and chi-square test was conducted to evaluate the differences between groups. We compared the difference with odds ratio (OR) and 95% confidence intervals (CI).

In univariate analysis, we use baPWV as dependent variable and other variables as independent variable to explore the relationship between them. In addition, in order to better investigate the association between UA and baPWV, we used the variation of regression coefficient and least absolute shrinkage and selection operator (lasso)^31,32^ regression analysis to screen covariates. In the subsequent model, we only retain the covariates that have more than 10% influence on the UA regression coefficient. In addition, LASSO regression is characterized by feature selection and complexity adjustment while fitting the generalized linear model. Consequently, whether the target variable is a continuous variable or a classification variable, it could be demonstrated and forecasted by LASSO regression. Feature selection refers to not setting variables into the model for matching, but discriminatingly placing variables into this pattern to acquire appropriate variables.

Furthermore, we used baPWV as dependent variable, UA as independent variable, and selected variables as covariates in multivariate regression analysis to adjust and observe the real association between UA and baPWV. Hosmer and Lemeshow test were used to measure goodness-of-fit. Considering that there is not necessarily a linear relationship between UA and baPWV in the real world, we use curve fitting to observe the change trend of UA and baPWV. In this study, we used SPSS 22 statistical software (SPSS, Chicago, IL, USA), R version 3.6.1 (https://www.r-project.org/) and EmpowerStats (http://www.empowerstats.com/cn/) for statistical analysis. P < 0.05 indicated that there was statistical difference (two sides).

## Result

### Baseline characteristics of four groups

**Figure 1:**
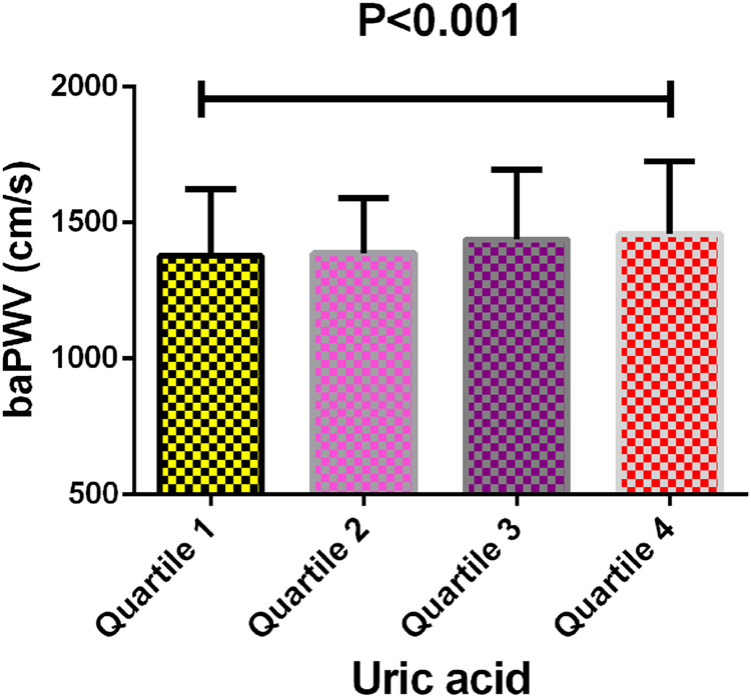
Comparison of baPWV in four groups.

The baseline characteristics of the four groups according to UA levels are shown in Table 1. Of 912 participants, 57.6% were male, with an average age of 51.5 ± 9.6 years. BMI, SBP, DBP, AST, ALT, Log2GGT, FBS, triglycerides, HDL, LDL, alcohol consumption, eGFR, sex, smoking status, ex-smoker and fatty liver of four groups were statistically different (all P < 0.05). No different was observed in age, TC, ABI, regular exerciser and menopausal status between the four groups (all P > 0.05). In addition, we found that baPWV (1377.60 ± 245.97, 1386.45 ± 202.76, 1437.91 ± 256.46 and 1457.94 ± 267.40, respectively) in the four groups had statistical difference, that is, UA level increased with the increase of baPWV (Fig. 1).

**Table 1:** Baseline characteristics of four groups.

Variable	Quantile 1	Quantile 2	Quantile 3	Quantile 4	P-value
N	216	240	198	258	
Age (year)	51.99 ± 9.05	50.86 ± 9.31	51.06 ± 10.12	50.72 ± 9.81	0.490
BMI (kg/m^2^)	21.86 ± 2.61	22.43 ± 2.80	23.77 ± 2.92*	24.34 ± 3.39*	<0.001
SBP (mmHg)	115.84 ± 14.46	118.01 ± 14.44	123.30 ± 15.75*	123.67 ± 13.95*	<0.001
DBP (mmHg)	72.26 ± 9.19	74.14 ± 9.92	78.27 ± 10.08*	79.61 ± 9.13*	<0.001
AST (U/L)	18.58 ± 5.34	19.82 ± 6.77	21.40 ± 9.87*	23.29 ± 8.89*	<0.001
ALT (U/L)	17.06 ± 6.64	20.15 ± 12.43	24.42 ± 15.60*	28.42 ± 17.00*	<0.001
Log2GGT (U/L)	3.93 ± 0.71	4.21 ± 0.74*	4.52 ± 0.87*	4.74 ± 0.83*	<0.001
FBS (mg/dl)	96.13 ± 20.74	97.26 ± 11.32	98.39 ± 10.66	100.13 ± 11.26*	0.014
TC (mg/dl)	208.01 ± 35.00	207.55 ± 35.69	212.34 ± 35.98	211.52 ± 37.00	0.388
Triglycerides (mg/dL)	69.62 ± 44.59	82.20 ± 51.46	113.55 ± 95.66*	131.13 ± 81.09*	<0.001
HDL (mg/dL)	59.50 ± 13.92	55.52 ± 14.94	51.67 ± 13.78*	48.12 ± 13.20*	<0.001
LDL (mg/dL)	123.86 ± 29.00	126.48 ± 30.58	131.24 ± 32.38*	130.60 ± 33.92	0.045
Alcohol consumption (g/week)	28.41 ± 77.69	45.50 ± 83.78	98.69 ± 155.45*	120.68 ± 164.88*	<0.001
eGFR (mL/min/1.73 m^2^)	73.98 ± 13.38	73.46 ± 10.59	69.41 ± 10.45*	65.36 ± 11.42*	<0.001
ABI	24.99 ± 174.20	54.78 ± 273.89	43.96 ± 246.06	92.42 ± 359.76*	0.057
baPWV	1377.60 ± 245.97	1386.45 ± 202.76	1437.91 ± 256.46	1457.94 ± 267.40*	<0.001
Sex (male, %)	45 (20.83%)	135 (56.25%)	163 (82.32%)	249 (96.51%)	<0.001
Smoking status (current, %)	23 (10.65%)	51 (21.25%)	54 (27.27%)	69 (26.74%)	<0.001
Regular exerciser (yes, %)	54 (25.84%)	43 (18.07%)	35 (17.95%)	45 (17.72%)	0.095
Fatty liver (yes, %)	25 (11.57%)	55 (23.01%)	63 (31.82%)	122 (47.29%)	<0.001
Menopausal status (Postmenopausal, %)	80 (46.78%)	41 (39.05%)	14 (40.00%)	3 (33.33%)	0.545

Data is represented as mean ± standard deviation or number (%). *Means P < 0.05 compared with Quantile 1.

Uric acid:

Quantile 1: 2.00–4.10 mg/dL; Quantile 2: 4.20–5.20 mg/dL; Quantile 3: 5.30–6.00 mg/dL and Quantile 4: 6.10–9.80 mg/dL.

BMI = body mass index; SBP = systolic blood pressure; DBP = diastolic blood pressure; ALT = alanine aminotransferase; AST = aspartate aminotransferase; GGT = glutamyltranspeptidase; FBS = fasting blood glucose; TC = total cholesterol; HDL = high density lipoprotein; LDL-C = low density lipoprotein; eGFR = estimated glomerular filtration rate; ABI = ankle brachial index; baPWV = brachial-ankle pulse wave velocity.

### Univariate analysis related to baPWV

We use baPWV as dependent variable and other variables as independent variable to explore which variables are related to baPWV. Univariate analysis showed that sex (female vs male: OR = −49.44, 95% CI −82.79 to −16.09), age (OR = 12.95, 95% CI 11.50 to 14.39), SBP (OR = 8.43, 95% CI 7.51 to 9.35), DBP (OR = 11.29, 95% CI 9.87 to 12.71), AST (OR = 3.40, 95% CI 1.44 to 5.37), ALT (OR = 1.50, 95% CI 0.38 to 2.61), Log2GGT (OR = 55.95, 95% CI 37.38 to 74.51), FBS (OR = 4.21, 95% CI 3.10 to 5.31), TC (OR = 22.77, 95% CI 11.25 to 34.30), triglycerides (OR = 0.71, 95% CI 0.27 to 1.16), HDL (OR = 0.44, 95% CI 0.23 to 0.65), LDL (OR = −1.33, 95% CI −2.42 to −0.23), fatty liver (Moderate or severe vs none: OR = 93.74, 95% CI 59.03 to 128.46), eGFR (OR = −6.39, 95% CI −7.65 to −5.12), menopausal status (No vs postmenopausal: OR = 207.39, 95% CI 158.43 to 256.36) and UA (Quantile 2 vs Quantile 1: OR = 8.85, 95% CI −36.05 to 53.75; Quantile 3 vs Quantile 1: OR = 60.32, 95% CI 13.22 to 107.42; Quantile 4 vs Quantile 1: OR = 80.34, 95% CI 36.19 to 124.49) were associated with baPWV (Table 2).

**Table 2:** Univariate analysis related to baPWV.

	baPWV
**Sex (n, %)**	
Male	Reference
Female	−49.44 (−82.79, −16.09) 0.0038
Age (year)	12.95 (11.50, 14.39) <0.0001
BMI (kg/m^2^)	4.95 (−0.16, 10.06) 0.0579
SBP (mmHg)	8.43 (7.51, 9.35) <0.0001
DBP (mmHg)	11.29 (9.87, 12.71) <0.0001
AST (U/L)	3.40 (1.44, 5.37) 0.0007
ALT (U/L)	1.50 (0.38, 2.61) 0.0086
Log2GGT (U/L)	55.95 (37.38, 74.51) <0.0001
FBS (mg/dl)	4.21 (3.10, 5.31) <0.0001
TC (mg/dl)	22.77 (11.25, 34.30) 0.0001
Triglycerides (mg/dL)	0.71 (0.27, 1.16) 0.0017
HDL (mg/dL)	0.44 (0.23, 0.65) <0.0001
LDL (mg/dL)	−1.33 (−2.42, −0.23) 0.0176
**Smoking status (n, %)**	
None or past	Reference
Current	−0.16 (−39.02, 38.70) 0.9936
**Ex-Smoker (n, %)**	
No	Reference
Yes	25.42 (−6.52, 57.36) 0.1192
Alcohol consumption (g/week)	0.07 (−0.05, 0.19) 0.2837
**Regular exerciser (n, %)**	
No	Reference
Yes	16.65 (−23.21, 56.52) 0.4132
**Fatty liver (n, %)**	
None	Reference
Moderate or severe	93.74 (59.03, 128.46) <0.0001
eGFR (mL/min/1.73 m^2^)	−6.39 (−7.65, −5.12) <0.0001
**Menopausal status (n, %)**	
Postmenopausal	Reference
No	207.39 (158.43, 256.36) <0.0001
**Uric acid**	
Quantile 1	Reference
Quantile 2	8.85 (−36.05, 53.75) 0.6992
Quantile 3	60.32 (13.22, 107.42) 0.0122
Quantile 4	80.34 (36.19, 124.49) 0.0004

Data is represented as: OR (95% CI) P-value.

Uric acid:

Quantile 1: 2.00–4.10 mg/dL; Quantile 2: 4.20–5.20 mg/dL; Quantile 3: 5.30–6.00 mg/dL and Quantile 4: 6.10–9.80 mg/dL.

BMI = body mass index; ALT = alanine aminotransferase; AST = aspartate aminotransferase; FBS = fasting blood glucose; TC = total cholesterol; HDL = high density lipoprotein; LDL-C = low density lipoprotein; eGFR = estimated glomerular filtration rate; ABI = ankle brachial index; baPWV = brachial-ankle pulse wave velocity.

### Screening of covariates related to baPWV

**Figure 2:**
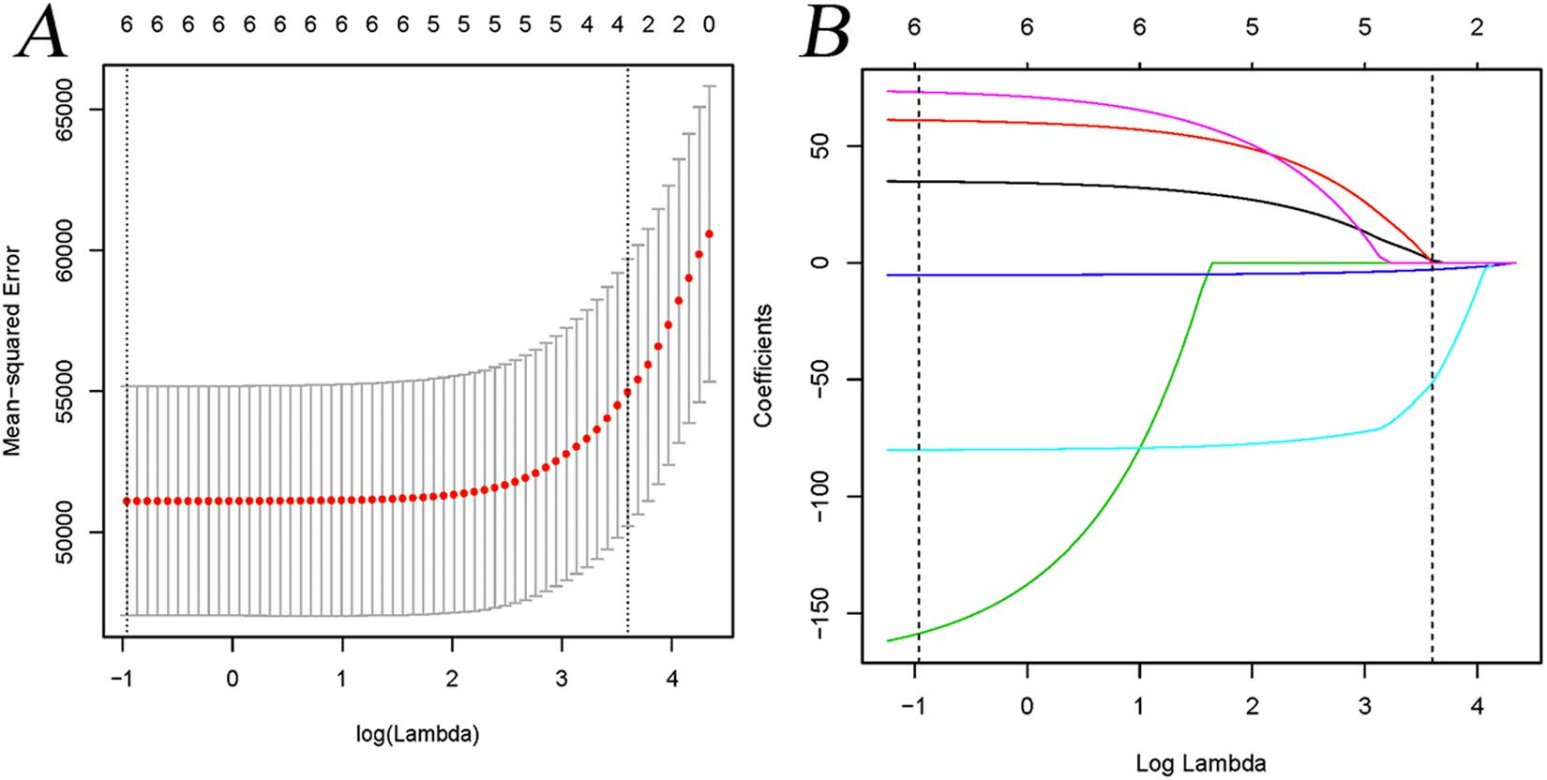
Lasso regression analysis of related factors with baPWV.

Firstly, considering that in clinical practice, SBP, DBP, AST, ATL and FBS have little or no clinical significance on baPWV, these five factors are excluded. Secondly, we use baPWV as dependent variable, UA as independent variable, and other variables with differences in univariate analysis are included in the regression analysis. Variables with more than 10% influence on the regression coefficient of UA will be included in the subsequent analysis. The result showed that log2GGT, fatty liver, EGFR and menopausal status were retained for subsequent analysis. In addition, we further used LASSO regression analysis to screen covariates. LASSO regression analysis showed that those four variables (log2GGT, fatty liver, EGFR and menopausal status) are retained and adjusted as final covariates in multivariate regression analysis (Fig. 2).

### Multivariate regression analysis of UA and baPWV

We used UA as independent variable, baPWV as dependent variable and log2GGT, fatty liver, EGFR and menopausal status as covariates to adjust in multivariate regression analysis to explore the association between UA and baPWV. In addition, in order to prove the stability of the results, three regression analysis models were developed. In model 1, none was adjusted and the result showed that UA is associated with baPWV (Quantile 2 vs Quantile 1: OR = 8.85, 95% CI −36.05 to 53.75; Quantile 3 vs Quantile 1: OR = 60.32, 95% CI 13.22 to 107.42; Quantile 4 vs Quantile 1: OR = 80.34, 95% CI 36.19 to 124.49). In model 2, fatty liver and menopausal status were adjusted and the result showed that UA is associated with baPWV (Quantile 2 vs Quantile 1: OR = 3.39, 95% CI −48.55 to 55.34; Quantile 3 vs Quantile 1: OR = 128.29, 95% CI 49.22 to 207.36; Quantile 4 vs Quantile 1: OR = 234.46, 95% CI 91.80 to 377.13). Similarly, this association still exists in model 3 (fatty liver, menopausal status, log2GGT and EGFR were adjusted) (Quantile 2 vs Quantile 1: OR = −9.92, 95% CI −60.16 to 40.32; Quantile 3 vs Quantile 1: OR = 82.34, 95% CI 4.00 to 160.68; Quantile 4 vs Quantile 1: OR = 143.13, 95% CI 0.75 to 285.51) (Table 3). In addition, Hosmer and Lemeshow test > 0.05, which indicates that the goodness-of-fit this model is good.

**Table 3:** Multivariate regression analysis of UA and baPWV.

Exposure	Model 1	Model 2	Model 3
Quantile 1	Reference	Reference	Reference
Quantile 2	8.85 (−36.05, 53.75) 0.6992	3.39 (−48.55, 55.34) 0.8982	−9.92 (−60.16, 40.32) 0.6990
Quantile 3	60.32 (13.22, 107.42) 0.0122	128.29 (49.22, 207.36) 0.0016	82.34 (4.00, 160.68) 0.0402
Quantile 4	80.34 (36.19, 124.49) 0.0004	234.46 (91.80, 377.13) 0.0014	143.13 (0.75, 285.51) 0.0497

Data is represented as: OR (95% CI) P-value.

Outcome variable: baPWV.

Exposure variable: Uric acid:

Quantile 1: 2.00–4.10 mg/dL; Quantile 2: 4.20–5.20 mg/dL; Quantile 3: 5.30–6.00 mg/dL and Quantile 4: 6.10–9.80 mg/dL.

Model 1: none was adjusted.

Model 2: fatty liver and menopausal status were adjusted.

Model 3: fatty liver, menopausal status, log2GGT and EGFR were adjusted.

### Curve fitting of UA and baPWV

**Figure 3:**
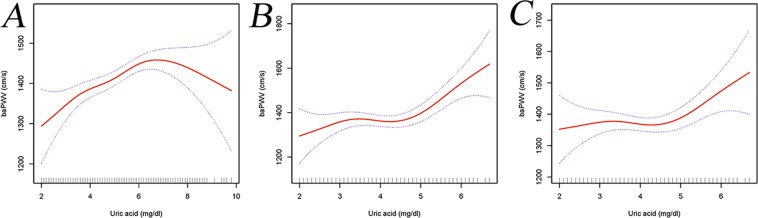
Curve fitting relationship between uric acid and baPWV. (**A**) none was adjusted. (**B**) fatty liver and menopausal status were adjusted. (**C**) fatty liver, menopausal status, log_2_GGT and EGFR were adjusted.

Curve fitting was used to examine the association between UA and baPWV. First, we did not adjust any variables, and then observed the curve relationship between UA and baPWV (Fig. 3A). Second, we adjusted fatty liver and menopausal status, and then observed the curve relationship between UA and baPWV (Fig. 3B). Finally, fatty liver, menopausal status, log2GGT and EGFR were adjusted, and then observed the curve relationship between UA and baPWV (Fig. 3C). Curve fitting showed that UA and baPWV had a non-linear positive correlation.

## Discussion

In this investigation, we found a considerable association between UA and baPWV. Despite the adjustment for potential confounding factors, the relationship is still significant (Quantile 2 vs Quantile 1: OR = −9.92, 95% CI −60.16 to 40.32; Quantile 3 vs Quantile 1: OR = 82.34, 95% CI 4.00 to 160.68; Quantile 4 vs Quantile 1: OR = 143.13, 95% CI 0.75 to 285.51). In addition, in further curve fitting, we also found that the relationship between UA and baPWV is not a simple linear relationship, but a nonlinear-positive correlation. Our results suggest that UA might be a useful predictor of atherosclerosis.

Few studies have paid close attention to the relationship between UA and baPWV, lacking theoretical basis to guide clinical practice. On the contrary, a series of studies have focused on the relationship between UA and atherosclerosis indicators^33–35^. A study included 1116 patients with suspected coronary heart disease (CHD) and divided them into four groups. The results showed that with the increase of UA level, the incidence of coronary atherosclerotic plaque and stenosis increased significantly (P < 0.05). In addition, elevated UA was associated with coronary artery calcification score^36^. Another study assessed the relationship between UA and subclinical atherosclerosis in Korean men. The results showed that after adjusting the known variables, the relationship between UA and coronary artery calcification (CAC) score and carotid intima-media thickness (cIMT) was OR = 1.101 (P = 0.046) and OR = 1.266 (P = 0.002), respectively^37^. Similarly, the CARDIA study intended to identify the association between UA and subclinical atherosclerosis in healthy adults. They found elevated UA was not related to BMI, but was related to the risk of coronary atherosclerosis. It is concluded that UA is an early biomarker of subclinical atherosclerosis, and this relationship is independent of BMI^38^. However, some researchers hold the opposite viewpoint. A cross-sectional study used Mendelian randomization to speculate the causal relationship between UA and atherosclerosis. It was found that the body mass index (BMI) alone could explain the changes in UA levels in women and men. After BMI or eGFR were adjusted, the relationship between UA and cIMT was not exist. The results showed that there was essentially a causal relationship between BMI and UA, BMI is a powerful confounding factor between UA and cIMT, which indicating that there was no evidence that UA played a role in the early stage of atherosclerosis^39^. In addition, a study based on the NHLBI database aims to explore the relationship between UA and CAC. The results showed that there was no evidence of a correlation between UA and CAC in both male and female (all P > 0.05), and this relationship did not exist in subgroup analysis, indicating that there was no evidence to confirm the relationship between UA and CAC. It is suggested that UA may not promote atherosclerosis through the effect of CAC^40^. Similarly, a cross-sectional study in Korea^29^ also showed that UA was not associated with baPWV levels. It should be noted that the participants in JH Lim study^29^ are between 20 and 80 years old, which may be the reason why the results are different from our study. In addition, population differences may also be the cause of different results.

Consistent with the studies of Sun Y^36^ and Zhang Z^37^, we explored the relationship between UA and baPWV based on data from a cross-sectional study and found that UA was positively correlated with baPWV, which was independent of BMI or eGFR. In addition, curve fitting displayed that the association between UA and baPWV is a non-linear positive correlation, not a simple linear relationship. It should be noted that our result is somewhat contrary to some predecessor’s result which have emphasized that no significant different was observed between UA and atherosclerosis. The possible explanations are as follows. First, in Oikonen M study^39^, the subjects were young people, aged between 30 and 45 years old, and is a study based on Finland population. In NHLBI study^40^, the average age of the subjects was 58 years old, and the subjects were the American population. Unlike previous studies, the subjects of our study were Japanese population aged between 24 and 84 years old. As is known to all, the degree of atherosclerosis tends to increase with age, and different dietary habits and genes in different populations can also lead to differences in atherosclerosis. Secondly, different measurement indicators will lead to different results. In our study, the evaluation index is baPWV; while in previous studies, cIMT or CAC were the indicators of evaluation. In addition, different measuring instruments could also lead to different results.

There are several aspects to explain the mechanism of uric acid and baPWV.

Hyperuricemia may form oxidized low-density lipoprotein (ox-LDL) by increasing oxygen free radicals^41–43^. Ox-LDL can not only promote the formation of foam cells, but also aggregate under the endothelium to form lipid stripes. Moreover, the apoptosis of intimal cells in coronary artery could be induced by activating multiple atherosclerotic pathways (ERK1/2 and bcl-2 pathways)^44–46^, and then leads to endothelial cell damage. In addition, oxygen free radicals and reactive oxygen species can also damage vascular endothelial function through leukocyte activation. Furthermore, urate crystals are deposited in the arterial wall, which induces inflammation, stimulates mast cells, induces lipid infiltration, and further damages the intimal cells of the arteries, thus blocking apolipoprotein B (apoB) metabolism, and ultimately leads to the aggravation of atherosclerosis^47,48^. Similarly, atherosclerosis can lead to glomerular hypoxia, and then increased lactic acid production, competitive excretion of uric acid, and increased UA levels.

### Strengths and limitations

This study has the following advantages. First of all, our study used univariate analysis, Lasso regression analysis and regression coefficient changes to screen covariates, and the results are more in line with clinical practice. Secondly, three regression models are used to adjust the potential variables in this study, and the results are consistent, which indicating that the results are stable. Thirdly, we use curve fitting to discover the real association between UA and baPWV. The result showed that the relationship between UA and baPWV is not a simple linear relationship, but a non-linear positive correlation, which is a remarkable difference from previous studies. It should be emphasized that this study has the following shortcomings. First, the current study is a second analysis based on a cross-sectional study, and cannot draw the causal relationship between UA and baPWV. Second, the participant of this study is the Japanese population, and the findings do not necessarily apply to other population. Third, we do not have access to the information about the participants’ medication or cardiovascular diseases, which might lead to mixed bias.

## Conclusions

Elevated UA were associated with baPWV, and this relationship is non-linear and positive correlation. suggesting that UA could be used as a predictor of atherosclerosis.
